# Endothelial glycocalyx degradation is associated with early organ impairment in polytrauma patients

**DOI:** 10.1186/s12873-021-00446-y

**Published:** 2021-04-20

**Authors:** Feng Qi, Hao Zhou, Peng Gu, Zhi-He Tang, Bao-Feng Zhu, Jian-Rong Chen, Jin-Song Zhang, Feng Li

**Affiliations:** 1 Emergency Intensive Care Unit, The Second Affiliated Hospital of Nantong University, Nantong First People’s Hospital, 6 Haier Xiang North Road, Nantong, 226001 Jiangsu China; 2 Department of Emergency Medicine, The First Affiliated Hospital of Nanjing Medical University, 300 Guangzhou Road, Nanjing, 210029 Jiangsu China

**Keywords:** Trauma, Glycocalyx, Syndecan-1, Heparan sulfate, Acute kidney injury, Trauma induced coagulopathy, Risk factor

## Abstract

**Background:**

Endothelial glycocalyx (EG) abnormal degradation were widely found in critical illness. However, data of EG degradation in multiple traumas is limited. We performed a study to assess the EG degradation and the correlation between the degradation and organ functions in polytrauma patients.

**Methods:**

A prospective observational study was conducted to enroll health participants (control group) and polytrauma patients (trauma group) at a University affiliated hospital between Feb 2020 and Oct 2020. Syndecan1 (SDC1) and heparin sulfate (HS) were detected in serum sample of both groups. In trauma group, injury severity scores (ISS) and sequential organ failure assessments (SOFA) were calculated. Occurrences of acute kidney injury (AKI), trauma-induced coagulopathy (TIC) within 48 h and 28-day all-cause mortality in trauma group were recorded. Serum SDC1 and HS levels were compared between two groups. Correlations between SDC1/HS and the indicators of organ systems in the trauma group were analyzed. ROC analyses were performed to assess the predictive value of SDC1 and HS for AKI, TIC within 48 h, and 28-day mortality in trauma group.

**Results:**

There were 45 polytrauma patients and 15 healthy participants were collected, totally. SDC1 and HS were significantly higher in trauma group than in control group (69.39 [54.18–130.80] vs. 24.15 [13.89–32.36], 38.92 [30.47–67.96] vs. 15.55 [11.89–23.24], *P* <  0.001, respectively). Trauma group was divided into high degradation group and low degradation group according to SDC1 median. High degradation group had more severe ISS, SOFA scores, worse organ functions (respiratory, kidney, coagulation and metabolic system), and higher incidence of hypothermia, acidosis and shock. The area under the receiver operator characteristic curves (AUC) of SDC1 to predict AKI, TIC occurrence within 48 h and 28-day mortality were 0.838 (95%*CI*: 0.720–0.957), 0.700 (95%*CI*: 0.514–0.885) and 0.764 (95%*CI*: 0.543–0.984), respectively.

**Conclusions:**

EG degradation was elevated significantly in polytrauma patients, and the degradation was correlated with impaired respiratory, kidney, coagulation and metabolic systems in early stage. Serum SDC1 is a valuable predictive indicator of early onset of AKI, TIC, and 28-day mortality in polytrauma patients.

## Background

Trauma remains a leading cause of morbidity and mortality worldwide and in China mainland [1, 2]. Organ function impairments are common in trauma, especially in polytrauma [3]. Endothelial glycocalyx (EG) is a layer of gel-like macromolecules widely distributed on the surface of vascular endothelium, mainly composed of proteoglycans (PGs) and glycosaminoglycans (GAGs). PGs, as the core proteins, are anchored on the surface of vascular endothelium, and the side chains of GAGs are covalently connected to it [4]. Highly sulfated GAGs side-chains are negatively charged and have electrical effects on plasma protein components such as albumin, fibrinogen, fibronectin, thrombomodulin, antithrombin-III, peroxidase, and cell adhesion molecules [5].

EG maintains vascular homeostasis by regulating vascular tension and permeability, inhibiting thrombosis in microvessels and regulating leukocyte adhesion on endothelial cell surface [6–8].Currently, EG abnormal degradations were widely detected in sepsis, tumor and other critical illness [9, 10]. However, data of EG degradation in polytrauma patients, and the association with early organ function impairment are obscure. PGs include SDC1, phosphatidylinositol PG, basal membrane PG, among which SDC1 is the main component [11]. GAGs include HS, hyaluronic acid (HA) and dermatan sulfates, HS accounts for more than 50% of GAGs [12].

We selected SDC1 and HS as the representation of EG degradation, and conducted this study to detect EG degradation in polytrauma patients, to fathom the correlation between EG degradation and early organ function impairment, and to demonstrate the predictive value of SDC1 and HS.

## Materials and methods

### Study design

A prospective observational study was designed to enroll polytrauma patients (trauma group) and healthy participants (control group) in a university affiliated hospital between February 2020 and October 2020. Trauma group inclusion criteria were: (1) two or more severe injuries caused by single reason in at least two areas of the body, (2) admitted to hospital after trauma less than 24 h. Exclusion criteria were: (1) age < 16 years, or > 75 years, (2) malignant tumor, (3) chronic hepatic or kidney diseases, (4) undrained pneumothorax. The study was approved by the Ethics Committee of the 2nd Affiliated Hospital of Nantong University (No.20190612), Jiangsu, China. The written informed consent was obtained from individual, patient or patient’s guardian.

### Data collection

Gender, age and body mass index (BMI) of both groups were documented. In trauma group, the reasons of injury were recorded, injury severity scores (ISS), sequential organ failure assessment scores (SOFA) were graded, occurrences of hypothermia (T < 35 °C), shock, acidosis, mechanical ventilation (MV), and traumatic brain injury (TBI) on admission were recorded, incidences of acute kidney injury (AKI), trauma induced coagulopathy (TIC) within 48 h, and 28-day mortality were documented.

### Laboratory methods

(1) Blood samples were collected on admission for trauma group. Blood samples of both groups were centrifuged (2500 g for 15 min at room temperature) and stored at − 80 °C. Double antibody sandwich ELISA tests were conducted by Enzyme calibration equipment (Thermo scientific Inc., Waltham, MA, USA), to detect SDC1 (EK1339, BOSTER Biological Tech., Wuhan, China), HS (E-EL-H2364c, Elabscience Biotechnology Co., Ltd., Wuhan, China), interleukin-6 (IL-6) (KE00139, Proteintech Group Inc., Rosemont, IL, USA), and tumor necrosis factor-α (TNF-α) (KE00068, Proteintech Group Inc., Rosemont, IL, USA), respectively. The tests were processed using unthawed samples within 6 months, and according to the manufacturer’s directions. All samples and standards were assayed in duplicate. (2) Standard laboratory tests were measured on admission in the clinical laboratory of the hospital as the following:①Blood routine (leukocyte count [WBC], hemoglobin [Hb], and platelet count [PLT]), Liver and kidney function (total bilirubin [TBIL], albumin [ALB], urea nitrogen [BUN], and serum creatinine [Cr], coagulation function (fibrinogen [Fib], prothrombin time [PT], activated partial thromboplastin time [APTT], anti-thrombin - [AT-III], and D-dimer), cardiovascular indicators (troponin I [Tn I], myoglobin [Mb], and N-terminal pro-B-type natriuretic peptide [NT-pro BNP]), and infection index (procalcitonin [PCT]), ②Blood gas analysis (pH, PaO_2_,PaO_2_/FiO_2_ ratio, HCO_3_^−^, Ca^2+^,and lactic acid [Lac]).

### Definitions of AKI and TIC

#### AKI definition

Serum Cr level increased ≥26.4 μmol/L compared with baseline, or increased over 1.5-times above baseline, or urine output < 0.5 ml/kg/h for at least 6 h [13].

#### TIC definition

Trauma induced prolonged coagulation time, PT > 18 s or APTT >60s [14].

### Semi-quantitative measurements of extravascular lung water (EVLW)

Lung ultrasound were performed using convex array (frequency 3.5–4.5 MHz, 2D mode, LOGIQ V1, GE Healthcare, Marlborough, MA, USA) to semi-quantitatively assess EVLW. Bilateral lungs were divided into eight regions [15],and the ultrasound clips were recorded. Each patient’s clips were independently graded and calculated average according lung ultrasound score (LUSS) [16] by two physicians with certifications of Chinese Critical Ultrasound Study Group (CCUSG). Regions scored according to the worst sign in the region (A lines or ≤ 2 B lines, score 0; ≥ 3 well-spaced B lines, score 1; coalescent B lines, score 2; tissue-like pattern, score 3).

### Statistical analyses

We calculated that a sample of 44 polytrauma patients would provide a power of 90% to detect the difference between variables and constants with effect size of 0.5, at a one-sided significance level of 0.025. A total sample size of 42 polytrauma patients would provide 80% power to detect an effect size of 0.8 between two groups, with a one-tailed significance level of 0.05. Statistical analyses were performed with SPSS 19.0 (IBM Inc., Chicago, IL, USA). Normality of continuous variables were detected by Shapiro-Wilk test. Normal distributed variable was presented as mean ***±*** standard deviation (mean ***±*** SD), and non-normal distributed variable as median (interquartile range) (median [IQR]), and categorical variable as number (percentage), respectively. Mann-Whitney *U* tests were performed for continuous variables comparisons between two groups. Categorical variables were compared by Fisher’s exact test. The correlation between the two variables was analyzed by Spearman’s correlation. The receiver operating characteristic (ROC) curves were graphed and area under the curve (AUC) were calculated to investigate the accuracy of indicators for predicting AKI, TIC within 48 h, and 28-day mortality in trauma group. All statistical tests were two-tailed, and *P* <  0.05 was considered statistically significant.

## Results

### Demographics

**Fig. 1:**
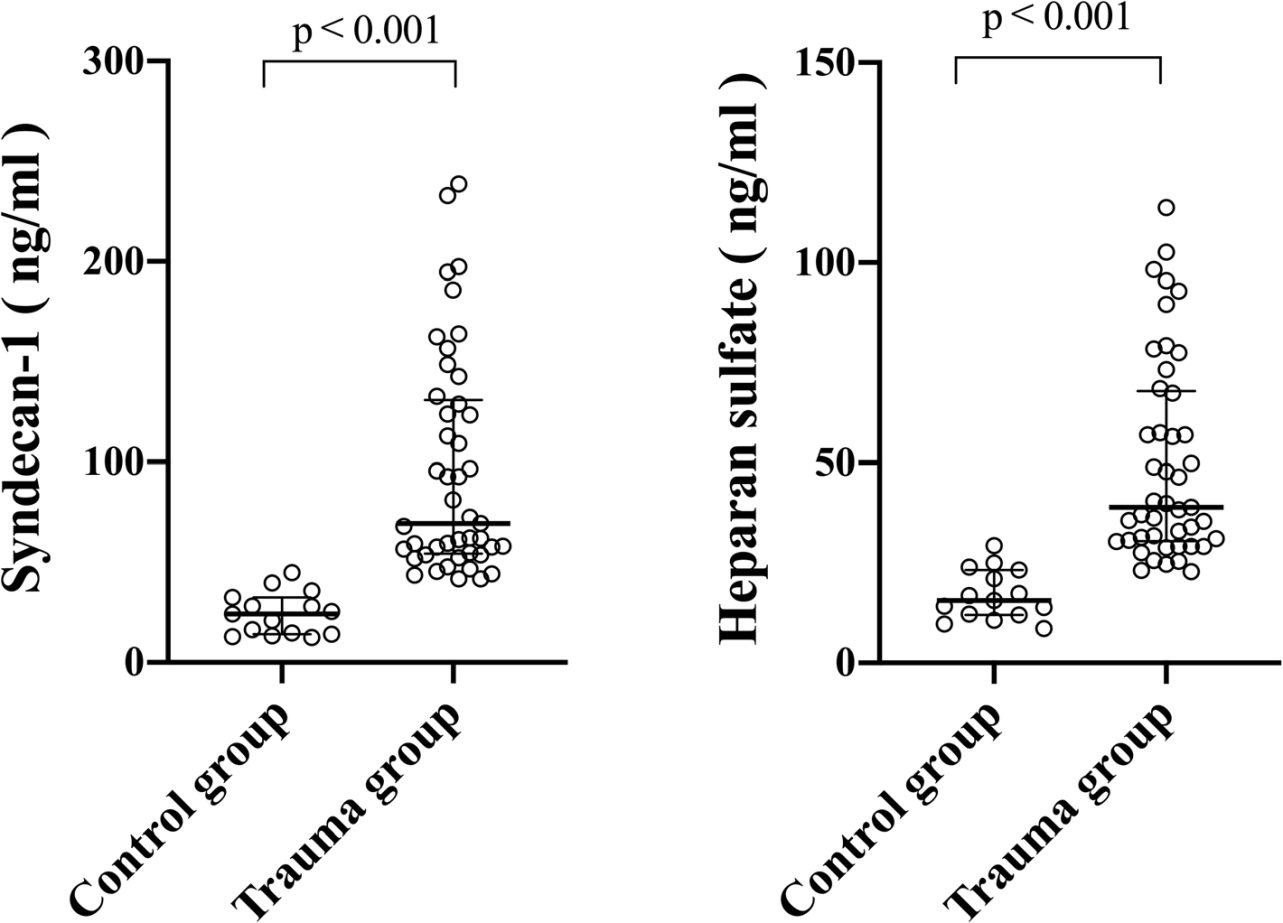
SDC1 and HS were significantly higher in trauma group compared with control group (69.39 [54.18–130.80] vs. 24.15 [13.89–32.36]; 38.92 [30.47–67.96] vs. 15.55 [11.89–23.24], *P* < 0.001 respectively)

The trauma group had a median ISS of 24 (17–29). Main causes of trauma were traffic injuries and falling injuries (53.3 and 31.1%, respectively). All polytrauma patients were admitted within 6 h after injury. There were 16 incidences of AKI, 13 incidences of TIC within 48 h, and 8 death cases within 28 days in trauma group, totally. Fifteen healthy participants were recruited as control group. There were none significant differences in age, gender and BMI between two groups (*P* > 0.05, respectively). The serum SDC1and HS in trauma group were significantly higher compared with control group (69.39 [54.18–130.80] vs. 24.15 [13.89–32.36]; 38.92 [30.47–67.96] vs. 15.55 [11.89–23.24]; *P* <  0.001, respectively) (Table 1, Fig. 1).

**Table 1:** Comparison of characteristics between trauma group and control group

Characteristic	Trauma group (***n =*** 45)	Control group (***n =*** 15)	***P*** value
**Demographic**			
**Age, mean** ***±*** **SD, year**	56.07 ***±*** 15.20	51.73 ***±*** 14.41	0.278
**Male, n (%)**	25 (73.3%)	9 (60.0%)	0.296
**BMI, mean** ***±*** **SD, kg/m**^**2**^	25.28 ***±*** 1.99	25.83 ***±*** 2.02	0.407
**EG degradation**			
**SDC1, median (IQR), ng/ml**	69.39 (54.18–130.80)	24.15 (13.89–32.36)	< 0.001
**HS, median (IQR), ng/ml**	38.92 (30.47–67.96)	15.55 (11.89–23.24)	< 0.001

Abbreviation: *BMI* body mass index; *EG* endothelial glycocalyx; *SDC1* syndecan-1; *HS* heparan sulfate

SD denotes standard deviation

IQR denotes interquartile range

### Correlation between EG degradation and organ function indicators

Spearman’s correlation analyses were conducted for EG degradation indicators (SDC1 and HS, respectively) with organ function indicators in trauma group, and found that serum SDC1 and HS were both positively correlated with PT, APTT, EVLW, Cr, NT-pro BNP, Mb, Lac, IL-6 and TNF-α, both negatively correlated with PaO_2_/FiO_2_ ratio, Ca^2+^, AT-III, pH and ALB (Table 2). Besides, SDC1 was significantly positively correlated with HS (*r* = 0.639, *P* <  0.001).

**Table 2:** Correlation between EG degradation indicators and organ function in trauma group on admission

Characteristic	Serum SDC1		Serum HS	
	*r*	*P* value	*r*	*P* value
**Demographic**				
**Age**	0.209	0.169	−0.009	0.956
**BMI**	−0.141	0.356	−0.024	0.875
**Score system**				
**GCS**	0.020	0.897	−0.312	0.037
**ISS**	0.380	0.010	0.320	0.032
**SOFA**	0.267	0.076	0.296	0.048
**Liver function**				
**TBIL**	−0.089	0.562	0.065	0.670
**ALB**	−0.652	< 0.001	−0.373	0.012
**Kidney function**				
**BUN**	0.558	< 0.001	0.262	0.082
**Cr**	0.603	< 0.001	0.388	0.009
**Respiratory function**				
**PaO**_**2**_	−0.318	0.033	−0.232	0.125
**PaO**_**2**_**/FiO**_**2**_ **ratio**	−0.458	0.006	−0.396	0.007
**EVLW**	0.515	< 0.001	0.344	0.021
**Metabolism system**				
**pH**	−0.513	< 0.001	−0.367	0.014
**HCO**_**3**_^**−**^	−0.471	0.001	−0.209	0.169
**Lac**	0.507	< 0.001	0.377	0.011
**Cardiovascular**				
**NT-pro BNP**	0.379	0.010	0.296	0.049
**Tn I**	0.289	0.054	0.289	0.054
**Mb**	0.481	0.001	0.455	0.002
**Coagulation**				
**Hb**	−0.468	0.001	−0.263	0.080
**PLT**	−0.314	0.036	−0.272	0.071
**Ca**^**2+**^	−0.545	< 0.001	−0.398	0.007
**Fib**	−0.006	0.970	0.037	0.809
**PT**	0.502	< 0.001	0.511	< 0.001
**APTT**	0.592	< 0.001	0.600	< 0.001
**AT-III**	−0.752	< 0.001	−0.522	< 0.001
**D-dimer**	−0.063	0.683	0.122	0.424
**Inflammation**				
**WBC**	−0.059	0.700	0.148	0.331
**PCT**	0.437	0.003	0.285	0.057
**IL-6**	0.384	0.009	0.326	0.029
**TNF-α**	0.531	< 0.001	0.390	0.008

Abbreviations: *EG* endothelial glycocalyx; *SDC1* syndecan-1; *HS* heparan sulfate; *BMI* body mass index; *GCS* Glasgow coma score; *ISS* injury severity score; *SOFA* sequential organ failure assessment; *TBIL* total bilirubin; *ALB* albumin; *BUN* blood urea nitrogen; *Cr* creatinine; *EVLW* extravascular lung water; *Lac* lactic acid; *NT-pro BNP* N-terminal pro-B-type natriuretic peptide; *Tn I* troponin I; *Mb* myoglobin; *Hb* hemoglobin; *PLT* platelet; *Fib* fibrinogen; *PT* prothrombin time; *APTT* activated partial thromboplastin time; *AT-III* anti-thrombin; *WBC* white blood cell; *PCT* procalcitonin; *IL-6* interleukin-6; *TNF-α* tumor necrosis factor

### Comparison within trauma group by different degree of degradation

Trauma group was divided into high degradation groups (SDC1 ≥ median) and low degradation groups (SDC1 < medians) by SDC1 median (69.39 ng/ml), comparing the differences between the two sets. The differences in general conditions and injury mechanisms were not statistically significant, and the high degradation group had higher ISS, SOFA scores, higher hypothermia, acidosis ratio, and higher inflammation indicators (IL-6, TNF-α), while respiratory (oxygen index, EVLW), liver (ALB), kidneys (BUN, Cr), bleeding and coagulation (Hb, PLT, Ca^2+^, PT, APTT, AT-III) and metabolic indicators (pH, HCO_3_^−^, Lac) were worse than the low degradation group (*P* <  0.05, respectively, Table 3). High EG degradation was associated with increased unadjusted odds of AKI (*OR* 2.870, 95%*CI* 1.089–7.562) and TIC (*OR* 3.188, 95%*CI* 1.009–10.072), but not 28-day mortality (*OR* 2.870, 95%*CI* 0.647–12.729).

**Table 3:** Comparison within trauma group deviated by degradation degree

Characteristic	Trauma group (n ***=*** 45)	EG degradation degree		***P*** value ^**§**^
		High degradation group (n = 23)	Low degradation group (n ***=*** 22)	
**SDC1, median (IQR), ng/ml**	69.39 (54.18–130.80)	128.74 (95.50–163.96)	54.17 (46.34–59.21)	< 0.001
**HS, median (IQR), ng/ml**	38.92 (30.47–67.96)	67.39 (48.90–89.58)	31.22 (27.07–36.44)	< 0.001
**Demographic**				
**Age, mean** ***±*** **SD, year**	56.0 ± 15.20	57.26 ***±*** 16.21	54.82 ***±*** 14.33	0.510
**Male, n (%)**	25 (55.56%)	12 (52.17%)	13 (59.09%)	0.641
**BMI, mean** ***±*** **SD, kg/m**^**2**^	25.27 ***±*** 1.99	24.94 ***±*** 2.06	25.61 ***±*** 1.91	0.212
**Cause of injury**				
**Traffic, n (%)**	22 (48.89%)	12 (52.17%)	10 (45.46%)	0.768
**Falling, n (%)**	14 (31.11%)	7 (30.43%)	7 (31.82%)	1.000
**Score system**				
**GCS, median (IQR)**	12.0 (6.0–15.0)	12.0 (5.0–15.0)	12.5 (6.0–15.0)	0.982
**ISS, median (IQR)**	24.0 (17.0–29.0)	26.0 (22.0–29.0)	17.0 (12.0–27.0)	0.011
**SOFA, mean** ***±*** **SD**	6.91 ***±*** 2.55	7.78 ***±*** 2.58	6.00 ***±*** 2.23	0.019
**TBI, n (%)**	21 (46.67%)	8 (34.78%)	13 (59.09%)	0.139
**Hypothermia, n (%)**	14 (31.11%)	12 (52.17%)	2 (9.09%)	0.003
**Acidosis, n (%)**	25 (55.56%)	18 (78.26%)	7 (31.82%)	0.003
**Shock, n (%)**	28 (62.22%)	18 (78.26%)	10 (45.45%)	0.033
**MV, n (%)**	36 (80.00%)	21 (91.30%)	15 (68.18%)	0.071
**AKI, n (%)**	16 (35.56%)	12 (52.17%)	4 (18.18%)	0.029
**TIC, n (%)**	13 (28.89%)	10 (43.48%)	3 (13.64%)	0.047
**28-d mortality, n (%)**	8 (17.78%)	6 (26.09%)	2 (9.09%)	0.243
**Organ function**				
**TBIL, median (IQR), μmol/L**	14.2 (11.2–22.3)	14.2 (10.9–22.1)	14.85 (11.35–22.55)	1.00
**ALB, median (IQR), g/L**	30.6 (21.9–37.3)	22.8 (19.3–32.5)	34.6 (30.58–41.33)	< 0.001
**BUN, median (IQR), mmol/L**	6.10 (5.05–9.00)	8.80 (5.70–11.20)	5.30 (4.55–6.30)	0.002
**Cr, median (IQR), μmol/L**	72.1 (52.3–103.3)	89.9 (62.5–144.0)	55.0 (46.73–75.85)	0.001
**PaO**_**2**_**, median (IQR), mmHg**	112.8 (89.5–155.1)	101.2 (86.7–134.4)	131.7 (92.1–168.7)	0.102
**PaO**_**2**_**/FiO**_**2**_ **ratio, mean** ***±*** **SD**	289.2 ***±*** 107.1	243.3 ***±*** 87.4	337.1 ***±*** 106.4	0.005
**EVLW, median (IQR)**	6.00 (4.25–9.50)	9.00 (6.00–10.50)	4.75 (2.88v6.63)	0.001
**Metabolism**				
**pH, mean** ***±*** **SD**	7.30 ***±*** 0.12	7.25 ***±*** 0.13	7.36 ***±*** 0.07	0.003
**HCO**_**3**_^**−**^**, median (IQR), mmol/L**	20.3 (15.8–22.4)	18.4 (14.2–20.7)	21.75 (19.65–23.10)	0.009
**Lac, median (IQR), mmol/L**	4.43 (2.65–7.99)	6.60 (4.21–9.60)	3.39 (2.26–4.57)	0.003
**Cardiovascular**				
**NT-pro BNP, median (IQR),**	265.3 (130.4–839.0)	723.6 (163.1–1136.0)	209.8 (119.1–430.2)	0.063
**Tn I, median (IQR), μg/L**	0.09 (0.03–0.26)	0.22 (0.01–1.14)	0.05 (0.03–0.17)	0.108
**Mb, median (IQR), μg/L**	652.0 (302.8–1141.0)	945.7 (631.2–1890.0)	466.0 (249.4–758.3)	0.010
**Bleeding and coagulation**				
**Hb, mean** ***±*** **SD, g/L**	105.22 ***±*** 20.36	95.61 ***±*** 18.98	115.27 ***±*** 16.86	0.001
**PLT, mean** ***±*** **SD, ×10**^**9**^**/L**	151.09 ***±*** 51.93	132.52 ***±*** 53.18	170.50 ***±*** 43.75	0.021
**Ca**^**2+**^**, median (IQR), mmol/L**	1.09 (1.04–1.15)	1.06 (0.99–1.11)	1.12 (1.09–1.19)	0.001
**Fib, median (IQR), g/L**	1.50 (1.13–1.89)	1.52 (0.90–2.12)	1.48 (1.28–1.81)	0.910
**PT, median (IQR), s**	13.2 (11.9–16.9)	14.2 (13.2–19.5)	12.2 (11.2–13.2)	< 0.001
**APTT, median (IQR), s**	31.7 (27.0–42.8)	37.0 (31.7–51.6)	28.0 (25.4–31.4)	< 0.001
**AT-III, mean** ***±*** **SD, %**	100.91 ***±*** 19.16	87.24 ***±*** 13.02	115.21 ***±*** 13.15	< 0.001
**D-dimer, median (IQR),**	15,645 (6494–43,654)	15,645 (5385–63,978)	15,977 (8311–36,585)	0.874
**Inflammation**				
**WBC, mean** ***±*** **SD, ×10**^**9**^**/L**	15.29 ***±*** 5.80	15.09 ***±*** 6.76	15.50 ***±*** 4.73	0.750
**PCT, median (IQR), ng/ml**	1.34 (0.41–5.38)	2.45 (0.52–10.20)	0.90 (0.23–2.05)	0.027
**IL-6, median (IQR), ng/ml**	281.71 (160.97–507.79)	351.49 (227.93–762.31)	234.56 (117.71–382.92)	0.023
**TNF-α, median (IQR), ng/ml**	70.23 (54.03–106.93)	95.32 (64.90–131.48)	61.88 (48.01–79.85)	0.002

Abbreviations: *SDC1* syndecan-1; *HS* heparan sulfate; *BMI* body mass index; *GCS* Glasgow coma score; *ISS* injury severity score; *SOFA* sequential organ failure assessment; *TBI* traumatic brain injury; *MV* mechanical ventilation; *AKI* acute kidney injury; *TIC* trauma induced coagulopathy; *TBIL* total bilirubin; *ALB* albumin; *BUN* blood urea nitrogen; *Cr* creatinine; *EVLW* extravascular lung water; *Lac* lactic acid; *NT-pro BNP* N-terminal pro-B-type natriuretic peptide; *Tn I* troponin I; *Mb* myoglobin; *Hb* hemoglobin; *PLT* platelet; *Fib* fibrinogen; *PT* prothrombin time; *APTT* activated partial thromboplastin time; *AT-III* anti-thrombin; *WBC* white blood cell; *PCT* procalcitonin; *IL-6* interleukin-6; *TNF-α* tumor necrosis factor

^**§**^ denotes the comparison between high degradation group and low degradation group

Indicates mortality rate for 28 days after admission

IQR denotes interquartile range

SD denotes standard deviation

Hypothermia denotes patient with temperature below 35 degree Celsius

Shock denotes patient with blood pressure maintained via infusions of dopamine, norepinephrine, epinephrine

### Predictive values of EG degradation indictors

**Fig. 2:**
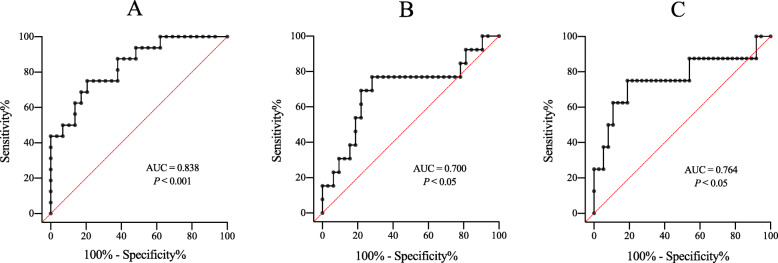
ROC curve showing the ability of the SDC1 to predict AKI, TIC within 48 h, and 28-day mortality in trauma group (**a**) AKI within 48 h, (**b**) TIC within 48 h, (**c**) 28-day mortality. Abbreviations: AKI, acute kidney injury; TIC, trauma induced coagulopathy; SDC1, syndecan-1. AUC denotes area under the curve

The ROC curve analyses indicated SDC1 was a valuable predicter to AKI (AUC of 0.838, cutoff of 94.12 ng/ml, sensitivity of 75%, and specificity of 79.3%), TIC (AUC of 0.700, cutoff of 92.66 ng/ml, sensitivity of 76.9%, and specificity of 71.9%) within 48 h, and 28-day mortality (AUC of 0.764, cutoff of 123.63 ng/ml, sensitivity of 75.0%, and specificity of 81.1%) (*P* <  0.05, respectively, Table 4, Fig. 2).

**Table 4:** Comparison of the predict value of SDC1, HS, ISS and SOFA on early AKI, TIC, and 28-day mortality

	AKI within 48 h			TIC within 48 h			28-day mortality		
	AUC	95% *CI*	*P* value	AUC	95% *CI*	*P* value	AUC	95% *CI*	*P* value
SDC1	0.838	0.720–0.957	< 0.001	0.700	0.514–0.885	0.038	0.764	0.543–0.984	0.021
HS	0.671	0.488–0.854	0.059	0.786	0.650–0.922	0.003	0.721	0.506–0.937	0.052
ISS	0.697	0.541–0.854	0.030	0.642	0.464–0.819	0.140	0.662	0.444–0.880	0.154
SOFA	0.635	0.463–0.806	0.138	0.662	0.506–0.818	0.091	0.583	0.406–0.760	0.467

Abbreviations: *AKI* acute kidney injury; *TIC* trauma induced coagulopathy; *SDC1* syndecan-1; *HS* heparan sulfate; *BMI* body mass index; *ISS* injury severity score; *SOFA* sequential organ failure assessment

AUC denotes area under the curve

## Discussion

Under physiological conditions, the synthesis and degradation of EG are in dynamic balance [5, 17]. At present, studies on abnormal EG degradation and impaired endothelial barrier have become an important direction of critical diseases [18, 19].EG abnormal degradation was found in sepsis [20], tumor [21], burns [22] and major surgery [23] patients. Previous studies have shown increased SDC shedding in traumatic brain injury and polytrauma patients [24], also the association of elevated serum SDC1 and higher ISS and 30-day mortality in trauma patients [25]. However, considering the complexity and heterogeneity of polytrauma, various factors, including major transfusions, emergency operations, nutrition insufficiency, infectious complications, et cetera, could influence the intra-hospital outcomes. Thus, research focused on the impact of elevated EG degradation on multiple organ functions, especially in the early stage, is necessary and meaningful. Our study confirmed the increased shedding of EG in polytrauma patients, and closely assessed the respiratory, coagulation, kidney, metabolism functions, and the activation of inflammation cytokines.

AKI has high morbidity and mortality in hospitalized patients, and various factors such as traumatic bleeding, fluid imbalance and inflammatory mediators are considered to be the potential pathogenesis of AKI [26]. EG coats the luminal surface of glomerular capillaries, therefore its integrity is essential for glomerular filtration barrier function. Study in mice showed the protection against glycocalyx HS degradation is associated with reduction of LPS-induced AKI [27]. In addition, tests in mice demonstrated inhibition of the syndecan-4 shedding process attenuated diabetes-induced kidney injury in the early stage [28]. It can be speculated that EG degradation contributes to glomerular filtration barrier dysfunction, which is the fundamental pathological character of AKI. In our study, SDC1 and HS were significantly correlated with renal function and metabolic indicators in trauma group patients, and the EG high degradation group had a higher incidence of AKI, indicating that abnormal EG degradation after trauma is a risk factor for early occurrence of AKI.

This study revealed SDC1 and HS were both correlated with blood loss and coagulation indexes including Hb, PLT, PT, APTT, AT-III and Ca^2+^. The high degradation group had significantly worse blood loss and coagulation indexes, and a higher incidence of TIC. SDC1 was a risk factor and predictive index for early TIC. Abnormal EG degradation may be involved in the pathophysiological process of TIC. Post-traumatic bleeding is the primary cause of death for trauma patients, and about one-third of trauma patients combined TIC, which significantly increased the risk of death [29]. The pathogenesis of TIC remains poorly understood. In the physiological condition, EG has the effects of inhibiting platelet adhesion on the endothelial surface, anti-microthrombosis and contributing to the balance between coagulation and anticoagulant. There have been several studies conducted in an attempt to identify the role of EG degradation in the mechanism of TIC. In ewe severe trauma model, EG shedding and activation of the protein C pathway were detected, and both correlated with the occurrence of TIC [30]. In vitro human whole blood study showed glycocalyx components exhibited inhibitory effects on platelet function, coagulation, and fibrinolysis [25]. EG degradation components are suggested as mediators in TIC, although the exact mechanism remains unclear. Researchers propose that post-traumatic bleeding, inflammation and abnormal EG degradation may participate in the TIC mechanism [31].

Due to the electrochemical properties of highly sulfated GAG side chain complex of EG, the permeability of EG to solute molecules is dependent on molecular size and its negative charge, which plays a role in isolating water and maintain the gel-like structure of EG, maintaining the low permeability of albumin and preventing the extravasation of intravascular liquid [12]. The increase of EVLW is the basic pathophysiological change in the early stage of lung injury. Studies of isolated animal lungs and isolated human donor lungs indicated that LUSS [32] can accurately reflect the degree of extravascular lung water [33, 34], and more clinical evidence supported the application of lung ultrasound in critical diseases [35]. This study found that SDC1 and HS were both positively correlated with EVLW and negatively correlated with oxygenation index. Compared with the low degradation group, high degradation group had higher EVLW and lower oxygenation index, suggesting that EG degradation was related to early lung injury after trauma. The integrity of EG barrier structure and function is of great significance for the study of lung injury mechanism and lung protection after trauma [36].

This study showed that SDC1 and HS were significantly positively correlated with NT-pro BNP and Mb, but cardiac function after trauma was not evaluated, so the significance was not analyzed.

Studies have shown that abnormal expressions of IL-6, IL-8 and TNF-α may be involved in EG degradation [19, 23]. We observed serum IL-6 and TNF-α levels were significantly elevated in trauma group, and the levels were both positively correlated with SDC1 and HS levels, suggesting that post-traumatic inflammatory responses may be involved in the abnormal EG degradation mechanism, although various pathways could be associated with EG degradation, theoretically.

It should be noted that there were also some detects in our study. First, the selected cases were patients with polytrauma admitted to EICU, which may have selection bias and information bias. Second, the sample size that is small increases the likelihood of type II error, and decreases the power of the study. In consideration of the complexity of polytrauma, meanwhile, transfusion and emergency surgery further increase the heterogeneity of the research objects, enhancing sample size for subgroup analysis and observing longer periods would be more meaningful to demonstrate the mechanism and influence of EG degradation in polytrauma patients.

## Conclusions

In this prospective observational study, we found a significant elevated EG degradation in polytrauma patients and the association between EG degradation indicators and multiple organ function impairment, including coagulation, kidney, metabolism and respiratory system. Our study indicated SDC1 is a valuable predicter of early TIC, AKI and 28-day mortality in polytrauma patients.

## Data Availability

All data generated or analyzed during this study are including in this article.

## Abbreviations

APTT: Activated partial thromboplastin
AKI: Acute kidney injury
ALB: Albumin
AT-III: Anti-thrombin-III
AUC: Area under the curve
BUN: Blood urea nitrogen
BMI: Body mass index
CCUSG: Chinese Critical Ultrasound Study Group
Cr: Creatinine
EG: Endothelial glycocalyx
ELISA: Enzyme-linked immunosorbent assay
EVLW: Extravascular lung water
Fib: Fibrinogen
GCS: Glasgow coma score
GAG: Glycosaminoglycan
Hb: Hemoglobin
HS: Heparan sulfate
ISS: Injury severity score
IL-6: Interleukin-6
IQR: Interquartile range
Lac: Lactic acid
WBC: Leukocyte count
LUSS: Lung ultrasound score
MV: Mechanical ventilation
Mb: Myoglobin
NT-pro BNP: N-terminal pro-B-type natriuretic
PLT: Platelet
PCT: Procalcitonin
PG: Proteoglycan
PT: Prothrombin time
ROC: Receiver operating characteristic
SOFA: Sequential organ failure assessment
SD: Standard deviation
SDC1: Syndecan-1
TBIL: Total bilirubin
TIC: Trauma induced coagulopathy
TBI: Traumatic brain injury
Tn I: Troponin I
TNF-α: Tumor necrosis factor-α
